# Efficient error correction for next-generation sequencing of viral amplicons

**DOI:** 10.1186/1471-2105-13-S10-S6

**Published:** 2012-06-25

**Authors:** Pavel Skums, Zoya Dimitrova, David S Campo, Gilberto Vaughan, Livia Rossi, Joseph C Forbi, Jonny Yokosawa, Alex Zelikovsky, Yury Khudyakov

**Affiliations:** 1 Laboratory of Molecular Epidemiology and Bioinformatics, Division of Viral Hepatitis, Centers for Disease Control and Prevention, 1600 Clifton Road NE, 30333 Atlanta, GA, USA; 2 Laboratório de Virologia, Instituto de Ciências Biomédicas, Universidade Federal de Uberlândia, Uberlândia 38400-902, Brazil; 3 Department of Computer Science, Georgia State University, 34 Peachtree str., 30303, Atlanta, GA, USA

## Abstract

**Background:**

Next-generation sequencing allows the analysis of an unprecedented number of viral sequence variants from infected patients, presenting a novel opportunity for understanding virus evolution, drug resistance and immune escape. However, sequencing in bulk is error prone. Thus, the generated data require error identification and correction. Most error-correction methods to date are not optimized for amplicon analysis and assume that the error rate is randomly distributed. Recent quality assessment of amplicon sequences obtained using 454-sequencing showed that the error rate is strongly linked to the presence and size of homopolymers, position in the sequence and length of the amplicon. All these parameters are strongly sequence specific and should be incorporated into the calibration of error-correction algorithms designed for amplicon sequencing.

**Results:**

In this paper, we present two new efficient error correction algorithms optimized for viral amplicons: (i) k-mer-based error correction (KEC) and (ii) empirical frequency threshold (ET). Both were compared to a previously published clustering algorithm (SHORAH), in order to evaluate their relative performance on 24 experimental datasets obtained by 454-sequencing of amplicons with known sequences. All three algorithms show similar accuracy in finding true haplotypes. However, KEC and ET were significantly more efficient than SHORAH in removing false haplotypes and estimating the frequency of true ones.

**Conclusions:**

Both algorithms, KEC and ET, are highly suitable for rapid recovery of error-free haplotypes obtained by 454-sequencing of amplicons from heterogeneous viruses.

The implementations of the algorithms and data sets used for their testing are available at: http://alan.cs.gsu.edu/NGS/?q=content/pyrosequencing-error-correction-algorithm

## Background

Recent advances in the next-generation sequencing (NGS) methods allow for analyzing the unprecedented number of viral variants from infected patients and present a novel opportunity for understanding viral evolution, drug resistance and immune escape [1,2]. However, the increase in quantity of data had a detrimental effect on quality of reads. In the case of 454 GS-FLX titanium pyrosequencing, the mean error rate is 1.07% and the error-free haplotypes represent 10.09% - 67.57% of the total number of reads, depending on the read length [3]. Originally, the emphasis was on obtaining the consensus sequence, provided that the depth of coverage easily allowed for retrieving the main true sequence and its most common polymorphisms irrespective of the suboptimal quality of numerous individual reads. However, analysis of viral amplicons is usually applied to biological tasks requiring in-depth characterization of viral populations and entails the examination of individual error-free reads rather than consensus sequences.

The main purpose of an error correction algorithm for viral amplicons is to discriminate between artifacts and actual sequences. This task becomes especially challenging when applied to recognizing and preserving low-frequency natural variants in viral population. Currently, the most used error correction algorithms involve the clustering of reads [2,4,5]. PyroNoise clusters the flowgrams using a distance measure that models sequencing noise [4], whereas SHORAH clusters the reads in Bayesian fashion using the Dirichlet process mixture [5,6]. Other approaches to error correction are based on the use of multiple sequence alignments [7] and k-mers, or substrings of reads of a fixed length k [8-10]. K-mer based algorithms are efficient but rather time and memory consuming. Additionally, these algorithms are prone to introduction of errors during the correction phase [5]. To overcome these disadvantages, the authors of EDAR algorithm [11] developed an approach for the detection and deletion of sequence regions containing errors. However, although this error deletion approach is efficient for shotgun sequencing, it is unacceptable for treatment of short amplicon reads commonly analyzed in viral samples due to their lower k-mer diversity.

The aforementioned methods are optimized for shotgun analysis and assume that the errors introduced by sequencing are randomly distributed. However, a recent assessment of the accuracy and quality of amplicon reads obtained using 454-sequencing showed that the error rate is not randomly distributed; rather, it is strongly affected by the presence of homopolymers, position in the sequence, size of the sequence and spatial localization in PT plates [3]. These findings indicate that many of these sequencing errors are sequence specific and may variably contribute to accuracy of reads generated from amplicons of different sequences. More importantly, the accuracy of amplicon sequencing should be improved by incorporating the factors affecting the error rate into calibration of the error correction algorithms.

In this paper, we present two new efficient error correction algorithms optimized for viral amplicons. The first algorithm (ET) includes a calibration step using sequence reads from single-clone samples. ET estimates an empirical frequency threshold for indels and haplotypes calculated from experimentally obtained clonal sequences, and also corrects homopolymer errors using sequence alignment. The second algorithm (KEC) does not need a calibration. It is based on the k-mer error correction. KEC optimizes the EDAR algorithm [11] for the detection of error regions in amplicons and adds a novel algorithm for error correction. Performance of both algorithms was compared to the clustering algorithm SHORAH using 24 experimental amplicon datasets obtained by 454- sequencing.

## Methods

### Experimental sequence samples

A set of 10 plasmid clones with different HCV HVR1 sequences was obtained. All the clones contained the modified HCV JFH1 sequence (GenBank accession number AB047639.1). Each of 10 HVR1 sequences was introduced into plasmid pJFH1 by two-step recombinant PCR using oligonucleotides encoding different HVR1 sequences, followed by digestion with a set of restriction endonuclease and ligation. Plasmids were then electroporated into *E. coli *and purified with the QIAGEN miniprep kit. All constructs were verified by DNA sequencing (BigDye v3.1 chemistry sequencing kit - Applied Biosystems, Foster City, CA) using an automated sequencer (3130xl Genetic Analyzer, Applied Biosystems). The average number of nucleotide differences among the 10 plasmid clones was 41.36 (minimum of 24 and maximum of 59).

A total of 24 samples of the plasmids were created, with 14 containing a single clone and 10 containing a mixture of clones in different concentrations (see Table 1). The junction E1/E2 region (309 nt) was amplified using a nested PCR protocol [12]. Briefly, all samples were amplified using the PerfeCTa SYBR FastMix chemistry (Quanta BioSciences, Gaithersburg, MD) and a set of external primers. The amplicons generated during the first round PCR were used as templates for a nested PCR using hybrid primer composed of the 454 primer adaptors, multiple identifiers and specific sequences complementary to the HCV genome. This allowed for multiplexing and downstream pyrosequencing procedure. Resulting amplicons were quantified using the Picogreen kit (Invitrogen, Carlsbad, CA). Integrity of each fragment was evaluated using Bioanalyzer 2100 (Agilent, Santa Clara, CA). PCR products were pooled and subjected to pyrosequencing using the GS FLX Titanium Series Amplicon kit in a 454/Roche GS FLX instrument. Sequencing of the reverse strand was conducted using the amp primer B. The initial reads were processed by matching to the corresponding identifier. Low quality reads were removed using the GS Run Processor v2.3 (Roche, 2010). The 454 run was divided in 4 sectors, two of which were used in the current experiment, one sector with a pool of the MID-separated single-clone samples and one sector with a pool of the MID-separated mixture samples (Table 1).

**Table 1:** Relative concentrations of the 10 clones in each sample and the number of raw reads obtained in the 454 experiment.

	1	2	3	4	5	6	7	8	9	10	N reads
**S1**	100	0	0	0	0	0	0	0	0	0	12096
**S2**	100	0	0	0	0	0	0	0	0	0	11134
**S3**	0	100	0	0	0	0	0	0	0	0	13183
**S4**	0	0	100	0	0	0	0	0	0	0	12080
**S5**	0	0	0	100	0	0	0	0	0	0	15507
**S6**	0	0	0	0	100	0	0	0	0	0	9643
**S7**	0	0	0	0	0	100	0	0	0	0	16215
**S8**	0	0	0	0	0	0	100	0	0	0	10583
**S9**	0	0	0	0	0	0	0	100	0	0	29101
**S10**	0	0	0	0	0	0	0	0	100	0	24230
**S11**	0	0	0	0	0	0	0	0	0	100	20560
**S12**	0	0	0	0	0	0	0	0	0	100	19133
**S13**	0	0	0	0	0	0	0	0	100	0	16542
**S14**	0	0	0	0	0	0	0	100	0	0	23629
**M1**	2.9	2.9	2.9	2.9	2.9	2.9	2.9	80.0	0	0	21168
**M2**	12.5	12.5	12.5	12.5	12.5	12.5	12.5	12.5	0	0	17577
**M3**	10.0	10.0	10.0	10.0	10.0	10.0	10.0	30.0	0	0	18482
**M4**	10.0	10.0	10.0	10.0	10.0	10.0	20.0	20.0	0	0	18722
**M5**	1.0	1.0	1.0	1.0	1.0	1.0	1.0	93.0	0	0	24519
**M6**	0.0	8.7	8.7	8.7	8.7	21.7	21.7	21.7	0	0	11561
**M7**	5.0	5.0	5.0	5.0	5.0	5.0	20.0	50.0	0	0	20931
**M8**	3.1	3.1	3.1	3.1	3.1	0.0	42.3	42.3	0	0	18733
**M9**	80.0	2.9	2.9	2.9	2.9	2.9	2.9	2.9	0	0	18007
**M10**	2.0	2.0	2.0	2.0	0.0	30.6	30.6	30.6	0	0	20677

### ET algorithm

The main purpose of the procedure is to calculate the frequency of erroneous haplotypes in amplicon samples where a single haplotype is expected. The calculation of an accurate threshold is dependent on high-quality pairwise sequence alignments and proper correction of homopolymers. The procedure was carried out with matlab [13] and involved the following steps:

(1) Amplicon size limits: All reads smaller than 90% of the expected amplicon length are deleted and all reads bigger than 110% of the expected amplicon length are clipped. All different haplotypes and their frequencies are calculated, which saves considerable time and memory at the following steps.

(2) Alignment to external references: Each haplotype is aligned against a set of external references of all known genotypes. For each haplotype the best match of the external set is chosen. The aligned sequence is clipped to the size of the chosen external reference.

(3) Alignment to internal references: The 20 most frequent haplotypes that do not create insertions or deletions in regard to the external reference are selected as the internal reference set. Each haplotype in the dataset is aligned against each member of internal references set. For each haplotype the best match of the internal set is chosen.

(3) Homopolymer correction: All homopolymers of 3 or more nucleotides are identified. If the homopolymer region includes an insertion, the nucleotide is removed. If the homopolymer includes a deletion, the gap is replaced by the missing nucleotide. Then all different haplotypes and their frequencies are recalculated.

(4) Outlier removal: All reads containing obvious PCR or sequencing artifacts are removed. Using the internal reference, the number of indels in each haplotype is found. An outlier threshold is defined for each file, calculated as the weighted average of the number of indels + 4 standard deviations. If a haplotype contains a number of indels higher than the outlier threshold, the haplotype is removed.

(5) Indel threshold: The read aligned to its reference is used to calculate the frequency of erroneous indels over all the 14 samples containing a single clone. An indel threshold is defined as the maximum frequency of erroneous indels (5.9%). If a haplotype contains an indel with a frequency lower than the indel threshold, the haplotype is removed.

(6) Haplotype error threshold: The frequency of erroneous haplotypes and its standard deviation is calculated over the 14 samples containing a single clone. A haplotype threshold was defined as the weighted average frequency of erroneous haplotypes + 9 standard deviations (0.40%). All haplotypes with a frequency lower than the haplotype threshold are removed.

(7) Removal of reads with Ns: All haplotypes with Ns are removed from the final file. This step was performed at the end rather than at the beginning to take advantage of the information that these reads provided regarding nucleotide frequencies at positions other than those with N.

### KEC algorithm

The scheme of KEC includes 4 steps:

(1) Calculate k-mers s and their frequencies kc(s) (k-counts). We assume that k-mers with high k-counts ("solid" k-mers) are correct, while k-mers with low k-counts ("weak" k-mers) contain errors.

(2) Determine the threshold k-count (error threshold), which distinguishes solid k-mers from weak k-mers.

(3) Find error regions. The error region of the read is the segment [i, j] such that for every p ∈ [i, j] the k-mer starting at the position p is considered weak.

(4) Correct the errors in error regions.

Let r = (r_1_, ..., r_n_), r_i_∈{A, T, G, C} be the fixed read. Denote by S_k_(i) the k-mer of r starting at the position i and by KC_k_(i) the k-count of this k-mer. For an arbitrary sequence s let pref_j _(s) be the prefix of length j of s.

#### (1) Calculating k-mers and k-counts

The unique reads r were stored together with their frequencies f_r_. The straightforward calculation of k-mers and k-counts is inefficient due to the usually large size of the data set. We use hash map, where each key is a k-mer s and the corresponding value is the array v(s) = ((r, i): s = S_k_(i) in the read r). The hash map can be rapidly constructed even for very large data sets.

#### (2) Finding the error threshold

The idea proposed in [8,9,11] is used to find the error threshold. Consider the distribution of frequencies of k-count values. Let f(v) be the frequency of the k-count value v. It is assumed, that k-counts of erroneous k-mers and correct k-mers follow different distributions (in [11] -- the exponential distribution and the series of Poisson models, in [8]-- Poisson distribution and Gaussian distribution, respectively). It was observed in [8,11], that it is not necessary to explicitly consider the model for the distribution, because the first minimum of f(v) satisfactorily separates different distributions, and therefore can be used as the error threshold. However, this approach often is not applicable to the amplicon data, because of the rather discrete distribution of k-count values than in the shotgun experiments. The first minimum of f(v) is usually equal to 0 and corresponds to the gap in the distribution (i.e. to the first k-count value, for which there is no corresponding k-mers). We define the end of the first sufficiently long segment of the consecutive 0's of f(v) as the error threshold t_er_. The length of the segment is the parameter of the algorithm.

#### (3) Finding error regions

The error regions in every read are calculated as follows. We first sequentially find isolated segments [i, j] such that for every p ∈ [i, j] KC_k_(p) ≤ t_er_. Then the k-mers of the read are clustered according to their k-counts using clustering by the variable bandwidth mean-shift method [14,15], as was proposed in [11]. We use the fast implementation FAMS [16] of the variable bandwidth mean-shift method. Finally, every segment is extended in both directions by adding consecutive positions q by the following rule: q is added if and only if there exists p ∈ [i, j] such, that k-mers S_k_(p) and S_k_(q) belong to the same cluster. Overlapping segments are joined, and the obtained segments are error regions.

#### (4) Error correction

This stage consists of 3 steps:

(4a) Error correction in "short" error regions (with lengths not exceeding k).

(4b) Error correction in "long" error regions (with lengths greater than k).

(4c) Haplotypes reconstruction and postprocessing.

Steps (4a) and (4b) could be used for any sequencing data and could be considered as the separate algorithm. Stage (4c) is designed for amplicon data.

##### (4a) Error correction in "short" error regions

(4a) is based on the following principle: correct the error in the read r by finding the minimum-size set of insertions, deletions and replacements, which transform the r into read with all k-mers being solid. This problem could be precisely solved by the dynamic programming [17], but this approach has two disadvantages: it is slow and the additional errors could be introduced. However, taking into account the homopolymer nature of errors and using the found error regions, the efficient and fast heuristics for the problem can be proposed.

We call error region x = [b, e] of a read r = (r_1_, ..., r_n_) a tail, if either b = 1 or e = n-k+1. Let l(x) be the length of x, and h_i_(w) denotes a sequence of i identical nucleotides w∈{A, T, G, C} (for i ≥ 2 h_i_(w) is a homopolymer).

Let us introduce the following notation for different types of single-nucleotide errors: Rep denotes replacement; Ins_1 _-- insertion, which does not create a homopolymer; Ins_p_, p ≥ 2, -- insertion, which creates a homopolymer of length p; Del_0 _-- deletion of an isolated nucleotide; Del_m _-- deletion from the homopolymer of length m+1, m ≥ 1. The straightforward verification shows, that the following proposition holds:

**Lemma 1**. *Suppose, that the non-tail error region x = [b, e] of the read r was caused by a one-nucleotide error E. Let w = r_e_*.

*1) If E = Rep, then l(x) = k*.

*2) If E = Ins_p_, 1 ≤ p ≤ k, then l(x) = k-p+1 and if p ≥ 2, the*n *x is followed by h_p-1_(w)*.

*3) If E = Del_m_, 0 ≤ m ≤ k, then l(x) = k-m-1 and if m ≥ 1, then x is followed by h_m_(c), where c≠w*.

According to Lemma 1, errors corresponding to non-tail error regions with lengths ≤ k could be identified and corrected. If the error region x = [b, e] of a read r = (r_1_, ..., r_n_) is a tail, then we delete from the read the suffix starting at the position b+k-1 (if e = n-k+1) or the prefix ending in the position e (if b = 0). This is the tail cutting operation. So, the basic scheme of the first stage of the error correction algorithm is the following.

**Algorithm 1**.

1) Consider every non-tail error region x = [b, e] with length not exceeding k of every read r = (r_1_, ..., r_n_). We assume, that x was caused by single isolated error at the position e. Taking into account the length of x and the sequence of nucleotides following it, identify the type of error using Lemma 1. If l(x) < k-1, then the type of error and its correction can be determined unambiguously. In the case of insertion remove r_e_; in the case of deletion duplicate r_e_, if it will introduce a solid k-mer. According to Lemma 1 the cases l(x) = k and l(x) = k-1 contains ambiguities. If l(x) = k, then x could be caused either by nucleotide replacement with 3 possible corrections or by simple nucleotide insertion. If l(x) = k-1 and r_e _= r_e+1_, then x could be caused either by the insertion of the nucleotide r_e _or by the deletion of the nucleotide c≠r_e _between r_e _and r_e+1_. Consider all possible corrections of the error and choose the correction, which introduce solid k-mer with the highest k-count.

2) Cut tails, delete short reads, recalculate k-mers and error regions, delete reads covered for more than 40% with error regions. Storing k-mers in the hash map allows to perform both steps 1) and 2) very fast.

3) Repeat steps 1) and 2) until there are no error regions in the data set or the fixed number of iterations is reached.

Algorithm 1 is heuristic. It assumes that errors in two consecutive nucleotides are extremely unlikely. There are elements of greedy strategy at different stages of the algorithm. Nevertheless, the disadvantages of greedy algorithms are less detrimental in real data. For example, during the consideration of error regions with lengths k and finding the possible replacement of the nucleotide r_e_, the existence of several different strong k-mers s with pref_k-1_(s) = (r_b_, ..., r_e-1_) (*) is possible. To find the replacement of r_e _Algorithm 1 will choose s with highest k-count, which is a greedy approach. However, the tests of Algorithm 1 on the selection of 24 different data sets with HCV sequencing data shows, that for the overwhelming majority of error regions of length k the strong k-mer s satisfying (*) is unique. The percentage of such error regions varies from 86 to 99.9% with average of 95.9%.

##### (4b) Error correction in "long" error regions

**Figure 1:**
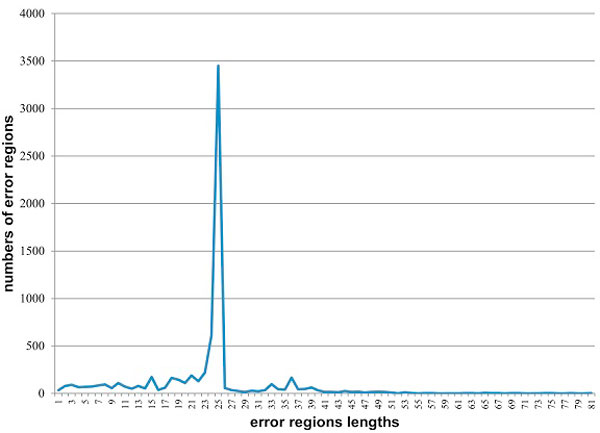
**Frequency distribution of error-region lengths in a sample of amplicon sequences (dataset M1, k = 25)**.

The error regions with lengths greater than k likely correspond to the situations when two or more errors are separated by less than k positions. This situation is significantly less probable than the presence of a single error. In Figure 1 the typical distribution of error regions lengths frequencies is illustrated. The number of "long" error regions is regulated by the parameter k. It should not be too small in order to obtain the more accurate boundary between strong and weak k-mers, and at the same time it should not be too large in order to better separate errors from each other. In our tests we used k = 25.

However, a certain number of "long" error regions is inevitable. We can locate the possible error bases for the error region x = [b, e], len(x) > k - it is the positions b+k-1 and e. However, for such error regions we lose the opportunity for prediction of the error type by combining their length and nucleotide sequence following it, because the length of error regions corresponding to the individual errors could not be determined.

There two ways to treat "long" error regions. One is to discard all reads with errors uncorrected by Algorithm 1. In this case, the error threshold and error regions are recalculated after finishing Algorithm 1, and reads containing error regions are discarded. The other way is to correct errors in "long" error regions. All possible errors at positions b+k-1 and e are considered to choose the correction procedure causing the introduction of k-mer with the highest k-count. Since these corrections are less reliable than for "short" error regions, correction of "long" error regions is conducted at the end of the algorithm after correcting "short" error regions, and Algorithm 1 is applied again before generating the final output of corrected reads. Both approaches are implemented in KEC, allowing users to exercise their preferences.

##### (4c) Haplotypes reconstruction and postprocessing

In the error-free data set of amplicon reads the collection of unique reads should be identical to the set of haplotypes, and the frequencies of unique reads should be proportional to the concentrations of haplotypes. Errors result in the increasing number of unique reads and divergence between the frequencies and concentrations.

The steps (4a) and (4b) dramatically reduce the numbers of unique reads in the data set. The corresponding statistics is presented in Table 2 for the data sets M1-M10. A set of corrected reads usually contains many error-free reads, which are subsequences of real haplotypes. Although such reads are not useful for finding true haplotypes, they are important for identifying haplotype frequencies. Therefore, the row "after (4a), (4b)" in Table 2 presents the number of unique reads and unique maximal reads (here and further by maximal reads we mean reads which are not subsequences of another reads).

**Table 2:** Number of reads in the datasets before and after steps (4a), (4b)

	**M1**	**M2**	**M3**	**M4**	**M5**	**M6**	**M7**	**M8**	**M9**	**M10**
Before (4a), (4b)	4220	4222	4418	4344	4426	3118	4661	4223	3986	4839
After (4a), (4b)	306/8	502/18	385/8	483/9	179/2	390/14	409/8	367/8	394/11	418/12

As illustrated in Table 2, some errors persist in the dataset after steps (4a), (4b). The small number of unique reads allows for using pairwise and multiple alignments to correct these errors. This idea is implemented in the following heuristic algorithm.

Let R = {r_1_, ..., r_n_} be a set of unique reads obtained after steps (4a),(4b), (f_1_, ..., f_n_) be the frequencies of these reads and R_max _⊆ R be a set of maximal reads of R. Let a_i, j _= 1, if the read r_j _is a subsequence of the read r_i_, and a_i, j _= 0, otherwise, i, j = 1, ..., n (by the definition a_i, i _= 1). Let also d_j _be the number of reads, which contain r_j _as the subsequence. The basic scheme of the error correction and haplotypes reconstruction algorithm is the following.

**Algorithm 2**.

1) For every r_i_∈R set its initial concentration $c_{i}:=f_{i}/\sum_{i=1}^{n}f_{i}$

2) Calculate set R_max_. For every r_i_∈R_max _recalculate its concentration by the following formula: $c_{i}:=\sum_{j=1}^{n}a_{i,j}\frac{c_{j}}{d_{j}}$

3) Calculate the multiple alignment of reads of R_max_. Identify homopolymer regions in the alignment. For every position of each homopolymer region calculate the total concentrations of reads with and without gap at this position. If both of these concentrations are non-zero and one of them is α times greater than the other, correct the position accordingly.

4) Put R: = R_max _and repeat 1) and 2). Calculate neighbor joining tree T of reads from R_max _based on their pairwise alignment score. Let P be the set of pairs of reads having common parents in T. For every (r_i_, r_j_) ∈P consider pairwise alignment of r_i _and r_j_. Identify homopolymer regions as in 3). Correct the position of the homopolymer region if and only if either the nucleotide difference between r_i _and r_j _is 1 or their concentrations differ more than α times.

5) Put R: = R_max _and repeat 1)-4) until no corrections can be made.

For the datasets described in this paper, α = 30 was used. ClustalW [18] was used for calculation of alignments and neighbor joining trees.

### Algorithm comparison

ET was implemented in Matlab and KEC in JAVA. Each sequence file was analyzed using ET, KEC and SHORAH error correction algorithms. SHORAH was applied several times using different parameters and the best attained results are reported here.

To evaluate performance of the three algorithms, nucleotide identity and frequency of the observed and expected true haplotypes were compared. Before doing this, the outputs of the three algorithms were post-processed to assure a fair comparison. The true haplotypes expected in each sample were aligned with the observed haplotypes using Needleman-Wunsch global alignment. The true haplotype with the best score was considered to be the match for each haplotype and the gapped ends were clipped in both sequences. For each method and sample, the following measures were calculated: (i) Missing true haplotypes, the number of expected haplotypes which were not found among the observed haplotypes; (ii) False haplotypes, the number of observed haplotypes with indels or nucleotide substitutions; (iii) Root mean square error, the disparity between the expected and observed frequencies of haplotypes; (iv) Average hamming distance, the distance between an observed false haplotype and its closest match, averaged over all false haplotypes.

## Results

### Error profile of single-clone samples

**Figure 2:**
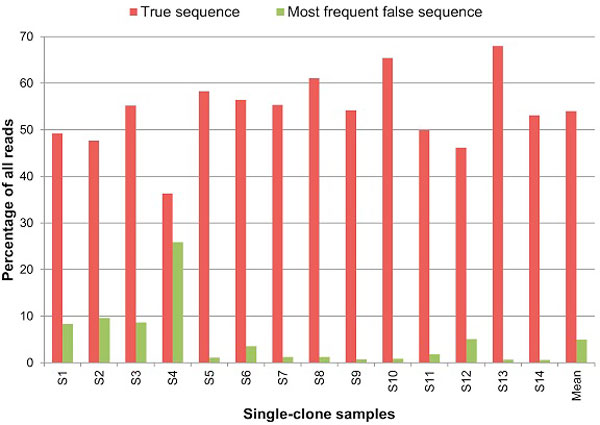
**Frequency of the true haplotype in single-clone samples**. Red bars show the percentage of all reads with the true haplotype and green bars show the frequency of the most common false haplotype.

The errors in reads of every single-clone sample were calculated by aligning each read with the corresponding clone sequence. The average percentage of error-free reads (true sequence) in the single-clone samples was 54.02% (Figure 2). The most common false haplotype was found with an average frequency of 4.96% but could be as high as 25.85% (homopolymer error in sample S4).

**Figure 3:**
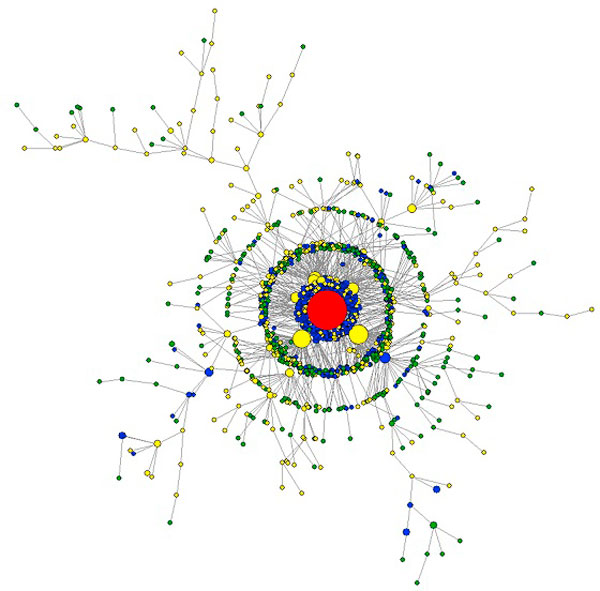
**Minimum spanning tree of single-clone sample S6**. Each node is a unique haplotype. The diameter of the node is proportional to the square root of its frequency. The true haplotype is shown in red, haplotypes with indel errors only are shown in yellow, haplotypes with nucleotide substitutions only are shown in blue and haplotypes with both types of errors are shown in green.

A minimum spanning tree (Figure 3) of a distance graph G_dist _of the dataset S6 illustrates the degree of sequence errors generated during 454-sequencing. This tree shows sequence relatedness of all unique haplotypes observed among the 454-reads of a single-clone sample. G_dist _is a complete weighted graph, the vertices of G_dist _are unique haplotypes, the weight of each edge h_1_h_2 _is the edit distance between h_1 _and h_2_. Most errors are found in low frequency haplotypes but homopolymer errors are usually found in high-frequency haplotypes.

**Figure 4:**
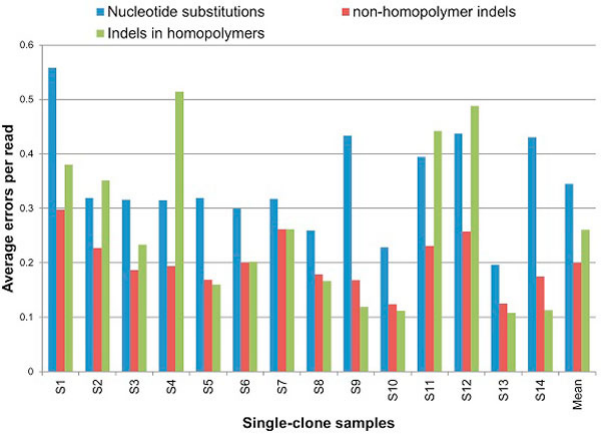
**Error profile of single-clone samples**. Three types of errors are shown: nucleotide replacements, non-homopolymer indels and indels in homopolymer.

Figure 4 shows the average number of errors per read, separating them according to their nature: nucleotide replacements, indels in homopolymers (deletion in a homopolymer or insertion which creates a homopolymer) and non-homopolymer indels. Most errors are insertions and deletions, 54.99% of which are located in homopolymers.

**Figure 5:**
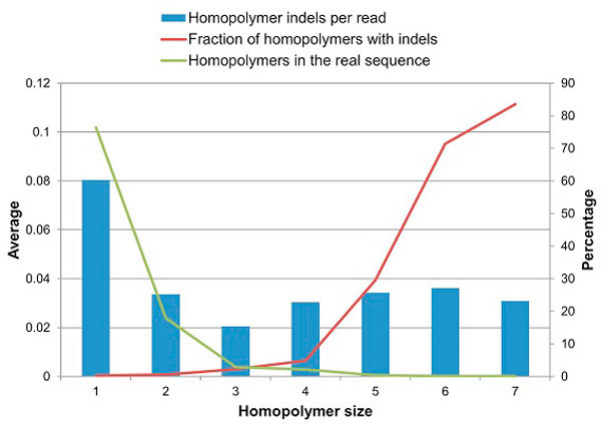
**Homopolymer indels distribution according to size**. (for the notation convenience we consider single nucleotides as homopolymers of length 1, so homopolymer indel in the homopolymer of length 1 is the insertion creating a homopolymer of length 2). Average homopolymer statistics over all 14 samples. The blue bars (left y-axis) show the number of homopolymer indels per read. The red line (left y-axis) shows the fraction of expected homopolymers of that size that contain errors. The green line (right y-axis) shows the percentage of homopolymers of that size that can be found in the real sequence.

Although the total number of nucleotide errors is high, they occur in different positions, making the frequency of individual reads with a particular error very low. The case with homopolymers errors is different, the longer a homopolymer is, the higher its fraction of errors (Figure 5). Small homopolymers (1 to 3) have high prevalence but low fraction of errors, whereas big homopolymers (4 to 7) have low prevalence but high fraction of errors.

### Algorithms comparison

Table 3 shows performance comparison of 3 algorithms applied to the experimental single-clone and mixture samples. Several modifications of the SHORAH parameters were used and the best results for this algorithm are shown in Table 3 (with SHORAH clustering hyper parameter equal to 0.1). Since SHORAH was presenting many false haplotypes, we attempted to improve its performance by using a frequency threshold, leaving only haplotypes with a frequency higher than 1%.

**Table 3:** Test results of the single-clone (S) and mixture (M) samples.

	ET				KEC				SHORAH_all				SHORAH_1%			
	**MT**	**FS**	**RMSE**	**HD**	**MT**	**FS**	**RMSE**	**HD**	**MT**	**FS**	**RMSE**	**HD**	**MT**	**FS**	**RMSE**	**HD**

S1	0	0	0.00	0.00	0	2	1.64	2.50	0	351	29.02	4.83	0	3	19.19	2.00
S2	0	0	0.00	0.00	0	0	0.00	0.00	0	269	30.12	4.44	0	3	21.70	1.00
S3	0	1	1.04	1.00	0	0	0.00	0.00	0	292	23.44	5.31	0	2	14.94	1.00
S4	0	1	0.96	2.00	0	0	0.00	0.00	0	271	44.68	5.37	0	1	37.87	1.00
S5	0	0	0.00	0.00	0	0	0.00	0.00	0	319	9.63	4.47	0	0	0.00	0.00
S6	0	1	0.70	2.00	0	0	0.00	0.00	0	194	18.70	3.90	0	3	12.03	1.00
S7	0	0	0.00	0.00	0	0	0.00	0.00	0	496	21.52	6.70	0	4	9.13	8.50
S8	0	0	0.00	0.00	0	0	0.00	0.00	0	262	14.37	4.58	0	1	3.16	1.00
S9	0	0	0.00	0.00	0	0	0.00	0.00	0	183	6.23	6.97	0	0	0.00	0.00
S10	0	0	0.00	0.00	0	0	0.00	0.00	0	288	7.77	5.11	0	0	0.00	0.00
S11	0	0	0.00	0.00	0	0	0.00	0.00	0	717	24.71	5.03	0	3	9.41	1.00
S12	0	1	0.65	2.00	0	0	0.00	0.00	0	611	25.94	5.52	0	4	10.61	1.50
S13	0	0	0.00	0.00	0	0	0.00	0.00	0	156	5.53	4.93	0	0	0.00	0.00
S14	0	0	0.00	0.00	0	0	0.00	0.00	0	161	6.83	6.60	0	0	0.00	0.00
Mean	0.00	0.29	0.24	0.50	0.00	0.14	0.12	0.18	0.00	326.43	19.18	5.27	0.00	1.71	9.86	1.29
M1	0	0	1.26	0.00	0	0	0.78	0.00	0	320	1.23	4.51	0	0	0.76	0.00
M2	0	0	1.50	0.00	0	0	1.95	0.00	0	738	3.70	4.44	0	2	3.47	1.00
M3	0	0	2.87	0.00	0	0	4.22	0.00	0	638	3.65	4.25	0	1	4.42	1.00
M4	0	0	2.12	0.00	0	0	3.09	0.00	0	577	2.88	5.20	0	1	3.08	1.00
M5	0	0	0.29	0.00	7	0	7.00	0.00	0	214	0.91	7.37	7	0	7.00	0.00
M6	0	0	2.45	0.00	0	0	1.81	0.00	0	394	3.25	4.50	0	0	2.57	0.00
M7	0	0	1.04	0.00	0	0	2.42	0.00	0	499	2.04	5.00	0	0	2.95	0.00
M8	0	0	0.40	0.00	0	0	2.25	0.00	0	336	3.30	5.48	0	1	3.22	1.00
M9	0	0	2.40	0.00	0	0	1.53	0.00	0	643	6.56	4.49	1	4	4.02	1.00
M10	0	0	3.70	0.00	0	0	4.16	0.00	1	637	6.13	5.30	1	2	5.73	1.50
Meann	0.00	0.00	1.80	0.00	0.70	0.00	2.92	0.00	0.10	499.60	3.37	5.05	0.90	1.10	3.72	0.65

MT: Missing true haplotypes; FS: False haplotypes; RMSE: root mean square error; HD: Average Hamming distance, averaged over all false haplotypes. SHORAH_all shows the raw results and SHORAH_1% the results based only on those haplotypes with a frequency > 1%.

**Figure 6:**
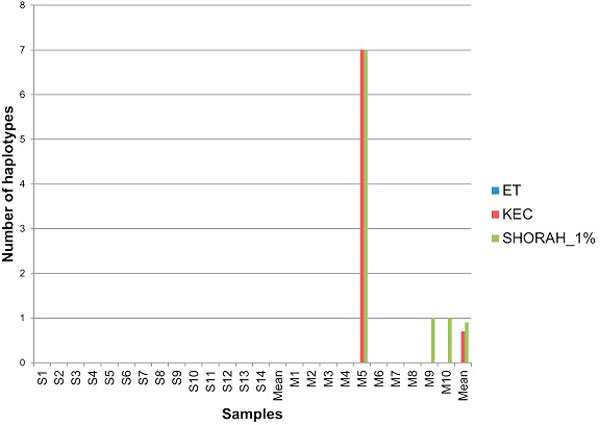
**Algorithm comparison: the number of missing true haplotypes**.

**Figure 7:**
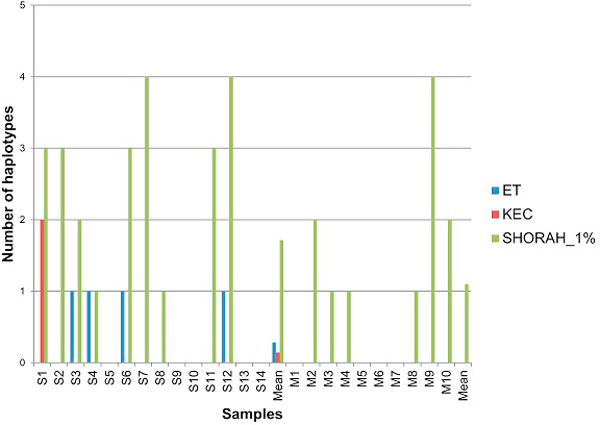
**Algorithm comparison: the number of false haplotypes**.

All methods found the correct sequence in each single-clone sample (Figure 6). When mixtures were tested, all three algorithms were successful in identifying most of the true haplotypes, with ET being the most sensitive. The major difference among algorithms is in the reported number of false haplotypes. ET and KEC reported a lower number of false haplotypes than SHORAH in every mixed sample (Figure 7).

**Figure 8:**
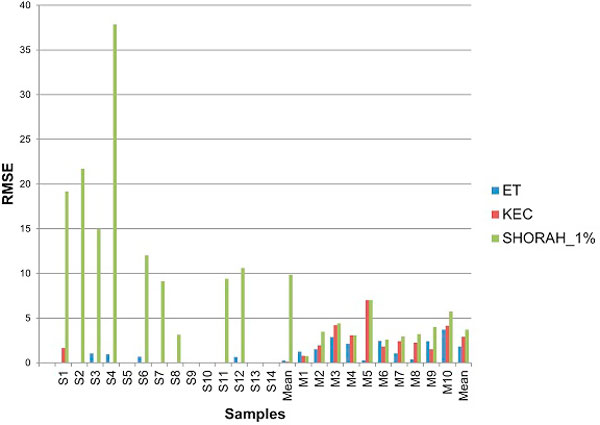
**Algorithm comparison: frequency of true haplotypes**.

The low Root mean square error (RMSE) between observed and expected frequencies of true haplotypes indicates that ET and KEC have a greater accuracy than SHORAH in single-clone samples. All three algorithms show equivalent results in the mixture samples (Figure 8). SHORAH is less accurate in identifying haplotype frequency owing to the large number of reported false haplotypes, presence of which reduces the observed frequency of the true haplotypes.

**Figure 9:**
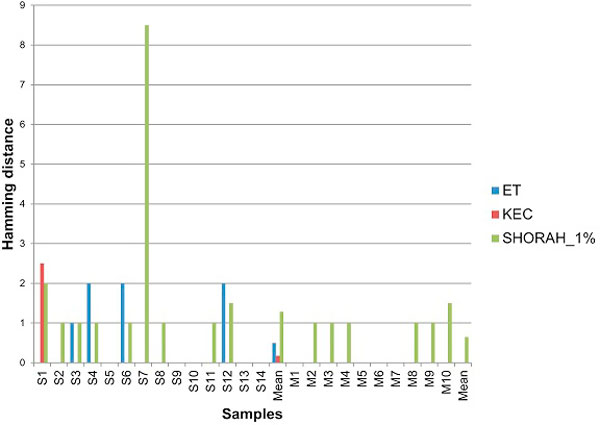
**Algorithm comparison: the average Hamming distance between false haplotypes and their true targets**.

Analysis of the Hamming distance between false haplotypes and their closest match shows that false haplotypes retained by KEC and ET are genetically closer to true haplotypes than the ones retained by SHORAH (Figure 9).

The test results of all samples are summarized in Table 3.

## Discussion

Hepatitis C virus (HCV) is a single-stranded RNA virus belonging to the *Flaviviridae *family [19]. HCV infects 2.2% of the world's population and is a major cause of liver disease worldwide [20]. HCV is genetically very heterogeneous and classified into 6 genotypes and numerous subgenotypes [21]. The most studied HCV region is the hypervariable region 1 (HVR1) located at amino acid (aa) positions 384-410 in the structural protein E2. Sequence variation in HVR1 correlates with neutralization escape and is associated with viral persistence during chronic infection [22-27]. NGS methods allow for analyzing the unprecedented number of HVR1 sequence variants from infected patients and present a novel opportunity for understanding HCV evolution, drug resistance and immune escape [1]. Most current methods are optimized for shotgun analysis and assume that the errors are randomly distributed. This assumption does not compromise the accuracy of shotgun sequencing as much as accuracy of amplicon sequencing. The sequencing error rate for amplicons is not randomly distributed [3] and should vary among amplicons of different primary structure.

In addition, current error-correction algorithms report performance measures related to their ability of finding true sequences, rather than the number of false haplotypes [1,2,4-6]. However, the biological applications of viral amplicons necessitate the use of error-free individual reads. All three methods studied here could find the correct sequences in both single-clone and mixture samples but showed marked differences in detecting the frequencies of the true haplotypes and the number of false haplotypes. We found that both ET and KEC are suitable for rapid recovery of high quality haplotypes from reads obtained by 454-sequencing.

The highly non-random nature of 454-sequencing errors calls for internal controls tailored to the amplicon of interest. The error distribution of single-clone samples helped us to calibrate the ET algorithm, thus facilitating its high accuracy in detection of true sequences in the HVR1 amplicons. ET was successful in finding the correct set of haplotypes in all 10 mixtures and in 10 of 14 single-clone samples, while found one false haplotype in 4 single-clone samples. KEC was correct for 13 of 14 single-clone samples (with 2 false haplotypes for one sample) and for 9 of 10 mixtures (not being able to find low-frequency clones in the mixture M5), having also the advantage that it does not need an experimental calibration step. SHORAH found all correct haplotypes for all single-clone samples and for 9 of 10 mixtures, having a very large number of false haplotypes and a significant divergence of expected and found frequencies. Introduction of a frequency cutoff for SHORAH results in loss of true haplotypes. SHORAH with frequency cutoff 1% was correct for 5 single-clone samples and for 3 mixtures, having both missing true and false haplotypes for other samples.

We highly encourage the sequencing of single-clone samples of the desired amplicon in order to understand the nature and distribution of the errors and to measure the performance of the algorithm in this particular amplicon.

Most algorithms are successful in identifying and removing low-frequency errors. However, reads with high-frequency homopolymer errors should not be removed but rather corrected, allowing for preservation of valuable data. All three algorithms correct reads with homopolymers in a different way. KEC uses the k-mer distribution to discern between erroneous and correct k-mers and then fixes homopolymers using a heuristic algorithm. ET fixes the homopolymers based on pairwise alignments with high-quality internal haplotypes. SHORAH clusters reads with a similar sequence, effectively creating a consensus haplotype. Sample S4 is particularly interesting because it included a false haplotype with a raw frequency of 25.85%. This false haplotype contained a deletion in a long homopolymer (n = 7). Both KEC and ET fixed this haplotype, but the clustering algorithm SHORAH preserved this false haplotype because of its high frequency and made it a center for the cluster of other reads, achieving a final frequency of 33.25%. The same situation occurs in most single-clone samples: in samples S1, S2, S3, S4, S6, S8, S11, S12 the second-frequent haplotypes with frequencies 13.3%, 16.2%, 11.6%, 33.25%, 5.2%, 2.79%, 2.9%, 3.89%, respectively differs from most frequent haplotype by one indel in a long homopolymer. The main assumption of clustering algorithms is that the observed set of reads represents a statistical sample of the underlying population and that probabilistic models can be used to assign observed reads to unobserved haplotypes in the presence of sequencing errors [5]. However, these algorithms assume that errors rates are low and randomly distributed, which is not true for the 454-sequencing of amplicons. Some homopolymer errors achieve very high frequencies, making very difficult to separate these false haplotypes from true ones using a clustering model.

## Conclusions

SHORAH, ET and KEC are equally accurate in finding true haplotypes. However, new algorithms, KEC and ET, are more efficient than SHORAH in removing false haplotypes and estimating the frequency of true ones. Both algorithms are highly suitable for rapid recovery of high quality haplotypes from reads obtained by NGS of amplicons from heterogeneous viruses such as HCV and HIV.

## Competing interests

The authors declare that they have no competing interests.

## Authors' contributions

PS developed and implemented the KEC algorithm. ZD and DSC developed the ET algorithm. ZD implemented the ET algorithm. YK and GV designed the experimental section. LR and JY created the plasmid clones. GV and JCF prepared the samples for sequencing. AZ helped to run the SHORAH software. PS, ZD and DSC analyzed all data. DSC, PS and YK wrote the manuscript. All authors read and approved the final manuscript.
